# Mapping and Analysis of Swi5 and Sfr1 Phosphorylation Sites

**DOI:** 10.3390/genes12071014

**Published:** 2021-06-30

**Authors:** Andrea Sevcovicova, Jana Plava, Matej Gazdarica, Eva Szabova, Barbora Huraiova, Katarina Gaplovska-Kysela, Ingrid Cipakova, Lubos Cipak, Juraj Gregan

**Affiliations:** 1 Department of Genetics, Faculty of Natural Sciences, Comenius University in Bratislava, Ilkovicova 6, 842 15 Bratislava, Slovakia; andrea.sevcovicova@uniba.sk (A.S.); jana.plava@savba.sk (J.P.); matej.gazdarica@img.cas.cz (M.G.); szabova.evka24@gmail.com (E.S.); barborahuraiova@gmail.com (B.H.); katarina.gaplovska@uniba.sk (K.G.-K.); 2 Cancer Research Institute, Biomedical Research Center of the Slovak Academy of Sciences, Dubravska Cesta 9, 845 05 Bratislava, Slovakia; 3 Institute of Molecular Genetics of the Czech Academy of Sciences, Videnska 1083, 142 20 Prague 4, Czech Republic; 4 Department of Genetics, Cancer Research Institute, Biomedical Research Center, Slovak Academy of Sciences, Dubravska Cesta 9, 845 05 Bratislava, Slovakia; ingrid.cipakova@savba.sk; 5 Advanced Microscopy Facility, VBCF and Department of Chromosome Biology, Max Perutz Labs, University of Vienna, Vienna Biocenter (VBC), Dr. Bohr-Gasse 9, 1030 Vienna, Austria

**Keywords:** Swi5, Sfr1, phosphorylation, DNA repair, recombination, meiosis, *Schizosaccharomyces pombe*

## Abstract

The evolutionarily conserved Swi5-Sfr1 complex plays an important role in homologous recombination, a process crucial for the maintenance of genomic integrity. Here, we purified *Schizosaccharomyces pombe* Swi5-Sfr1 complex from meiotic cells and analyzed it by mass spectrometry. Our analysis revealed new phosphorylation sites on Swi5 and Sfr1. We found that mutations that prevent phosphorylation of Swi5 and Sfr1 do not impair their function but *swi5* and *sfr1* mutants encoding phosphomimetic aspartate at the identified phosphorylation sites are only partially functional. We conclude that during meiosis, Swi5 associates with Sfr1 and both Swi5 and Sfr1 proteins are phosphorylated. However, the functional relevance of Swi5 and Sfr1 phosphorylation remains to be determined.

## 1. Introduction

Homologous recombination is a conserved process for repairing several types of lesions, including DNA double-strand breaks [1]. It is a precise DNA repair pathway, during which homologous DNA sequence is copied from an intact donor template. The ability to identify and exchange the strands of two homologous DNA molecules is mediated by RecA-family recombinases such as Rad51 and meiosis specific Dmc1, eukaryotic orthologs of the prokaryotic recombinase RecA. Rad51 and Dmc1 require several auxiliary factors in order to function properly. The Swi5-Sfr1 complex in the fission yeast *Schizosaccharomyces pombe* (Mei5-Sae3 in *Saccharomyces cerevisiae*) has been characterized as an auxiliary factor that stimulates Rad51 and Dmc1 activity. The Swi5-Sfr1 complex stabilizes Rad51 and Dmc1 filaments and stimulates their ATPase activity [2,3,4,5,6,7]. Swi5 in complex with Sfr1C (180 residue N-terminal deletion mutant of Sfr1) was successfully crystallized. Structural studies demonstrated that Swi5 and Sfr1C form a complex with a parallel coiled-coil heterodimer joined together via two leucine-zipper motifs and a bundle. Swi5-Sfr1C forms a kinked structure that is able to stimulate Rad51-mediated strand exchange. Docking of the atomic model of the Swi5-Sfr1C complex into the Rad51 filament model showed that the kinked structure fits well into the helical groove of Rad51 filament [8]. The N-terminal part of Sfr1 (Sfr1N) is intrinsically disordered and contains two sites that cooperatively bind Rad51, indicating that the primary function of Sfr1N is to mediate the interaction between Swi5-Sfr1 and Rad51 [6].

Swi5 and Sfr1 play an important role during meiosis, a specialized cell division that generates gametes with a haploid set of chromosomes from a diploid precursor [9]. This reduction in chromosome number results from one round of DNA replication followed by two nuclear divisions, meiosis I and meiosis II. During meiotic prophase I, homologous chromosomes pair and crossovers are created as one outcome of the repair of programmed DNA double-strand breaks via homologous recombination. Crossovers provide a physical connection between homologs, which is essential for their faithful segregation during the first meiotic division [10]. Mutation of either Swi5 or Sfr1 results in reduced homologous recombination in both mitotic and meiotic cells and elevated sensitivity to a number of DNA damaging agents, including ionizing radiation and methyl-methanesulfonate (MMS) [11,12,13,14,15].

The importance of homologous recombination is emphasized by its critical role during development and for tumor suppression. Defects in homologous recombination are associated with sensitivity to DNA damage, loss of genomic integrity and various diseases including Fanconi anemia and Bloom syndrome [16].

In this study, we investigated the role of post-translational modification by phosphorylation in the regulation of Swi5-Sfr1 function. We purified *S. pombe* Swi5-Sfr1 from meiotic cells and identified phosphorylation sites by mass spectrometry. We found that although the identified phosphorylation sites are not essential for the function of Swi5 and Sfr1, phosphomimetic *swi5* and *sfr1* mutants are only partially functional.

## 2. Materials and Methods

### 2.1. Strain Construction and General S. pombe Methods

*S. pombe* genetic procedures and growth media have been described by Forsburg et al., Moreno et al. and Phadnis et al. [17,18,19]. Deletions of *swi5* and *sfr1* genes were performed as described in Gregan et al. [20]. TAP-tagging was performed as described in Cipak et al. [21]. *S. pombe* strains were constructed by standard meiotic crosses, genotypes of strains used are listed in Table S3. Transformants were confirmed by PCR-based analysis and mutations of phosphorylation sites by nucleotide sequencing.

Plasmids pCloneHyg1-swi5-wt (p334), pCloneHyg1-swi5-S72AS84A (p335) and pCloneHyg1-swi5-S72DS84D (p336) were used to prepare JG17812, JG17813 and JG17814 strains, respectively. Plasmids pCloneHyg1-sfr1-wt (p320), pCloneHyg1-sfr1-13S-T/A (p321) and pCloneHyg1-sfr1-13S-T/D (p322) were used to prepare JG17984, JG17985 and JG17986 strains, respectively. *swi5* mutations were introduced into p335 and p336 plasmids using the QuikChange II Site-Directed Mutagenesis kit (Agilent Technologies, Santa Clara, CA, USA). Gene synthesis was used to prepare *sfr1* mutants in p321 and p322 plasmids (Integrated DNA Technologies, Coralville, IA, USA).

### 2.2. Microscopy

The immunofluorescence and microscopy techniques used to analyze chromosome segregation were performed as described in Rabitsch et al. [22].

To monitor the progression of meiosis, 1 ml aliquots of cultures were collected and fixed in 70% (*v*/*v*) ethanol. To visualize DNA, mounting medium with DAPI (Vectashield, Vector Laboratories, Burlingame, CA, USA) was used for microscopy (Zeiss Axio Imager Z2; Carl Zeiss AG, Oberkochen, Germany).

### 2.3. Spot Tests

YES plates containing methyl methanesulfonate (YES+MMS) were freshly prepared two days before the experiment. Cells were grown on YES plates for one day at 32 °C, resuspended in sterile water and cell concentration was determined using a Burker chamber. Cells were diluted in sterile water in 10-fold steps and 3–5 microliters of suspension was spotted onto standard YES and YES+MMS plates. The plates were incubated for 3–5 days at 30 °C.

### 2.4. Western Blot Analysis

Proteins were separated by electrophoresis through 12% polyacrylamide gels containing SDS (0.1%) and transferred to a PVDF membrane (G E Healthcare, North Richland Hills, TX, USA). The membrane was blocked with 5% (*w*/*v*) milk PBST (phosphate buffered saline buffer with 0.1% (*v*/*v*) Tween-20) and probed with primary antibodies. The TAP epitope was detected using PAP antibodies (rabbit antiperoxidase antibody linked to peroxidase) (Dako, Agilent Technologies, Santa Clara, CA, USA) at 1:10,000 dilution in 5% (*w*/*v*) milk PBST. Tubulin was detected using monoclonal anti-α-tubulin primary antibody produced in mouse (Sigma Aldrich, Merck, Darmstadt, Germany) at 1:10,000 dilution in 5% (*w*/*v*) milk PBST and rabbit anti-mouse HRP secondary antibody (Sigma Aldrich, Merck, Darmstadt, Germany) at 1:5000 dilution in PBST. Pierce ECL Plus Western Blotting Substrate (Thermo Fisher Scientific, Waltham, MA, USA) and Image Station 4000MM (Kodak, Rochester, NY, USA) were used for detection.

### 2.5. Protein Purification and Analysis

Induction of meiosis and monitoring of progression of meiosis were performed as described in Cipak et al. [23]. Meiotic cultures expressing Sfr1-TAP or Sfr1-5A-TAP were harvested around 3 hours after induction of meiosis and cells from fifteen-liter cultures were collected by centrifugation. The tagged proteins were isolated and analyzed by mass spectrometry as described previously in Cipak et al. [21].

## 3. Results and Discussion

### 3.1. Swi5 and Sfr1 Are Phosphorylated during Meiosis

In our previous work, we used the tandem affinity purification (TAP) protocol to purify Swi5-TAP and Sfr1-TAP from cycling *S. pombe* cells and found that Swi5 was phosphorylated on serine 72 and Sfr1 contained three phosphorylated serine residues (S26, S109 and S165) [24]. Additional phosphorylation sites on Sfr1 (S24, S26, T73, S109, S116 and S165) were published during the course of our work [25,26,27,28,29].

**Figure 1:**
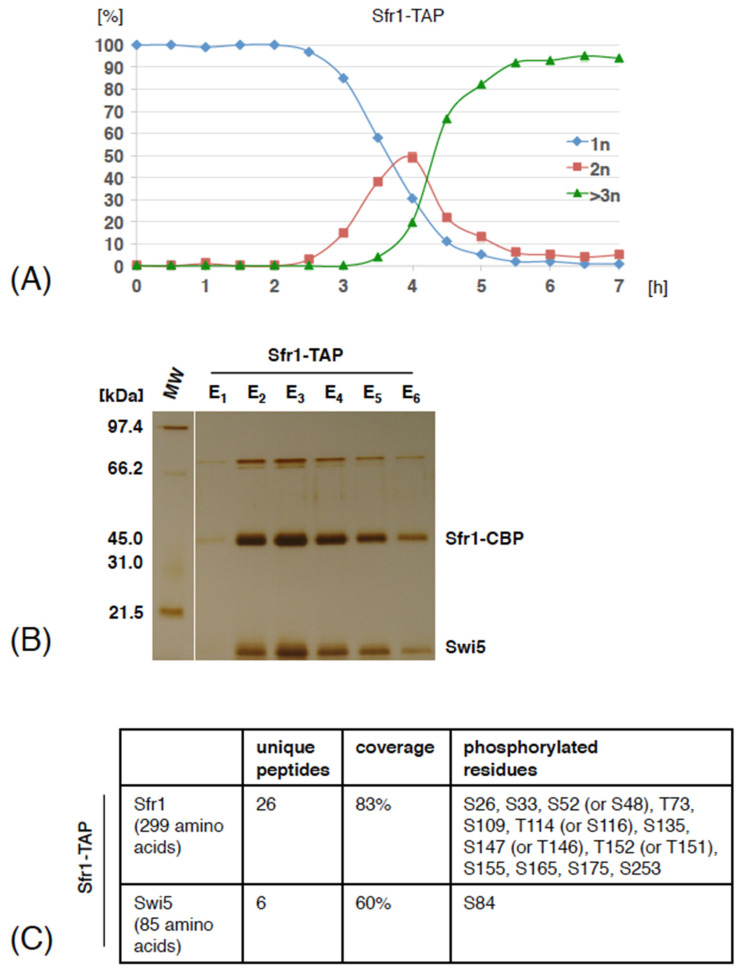
Swi5 and Sfr1 phosphorylation sites identified by mass spectrometry. (**A**) Diploid *pat1-114/pat1-114* cells expressing Sfr1-TAP were arrested by nitrogen starvation and released into meiosis at 34 °C by inactivation of Pat1. Small aliquots of cells were harvested at the indicated time points (hours). Fixed cells were stained with DAPI and nuclei were counted in 100 cells per time point. Shown are the fractions of cells that contained one nucleus (1n), two nuclei (2n) or more than two nuclei (>3n) at the indicated time points. (**B**) The cells were harvested around 3 hours after meiosis induction and Sfr1-TAP was isolated by tandem affinity purification. Purified proteins were separated on an SDS-PAGE gel and visualized by silver staining. Molecular weight marker (MW) is indicated on the left. Positions of Sfr1-CBP and Swi5 are indicated according to their predicted molecular weight. In parallel, elution E3 was subjected to analysis by mass spectrometry. (**C**) Swi5 and Sfr1 phosphorylation sites identified by mass spectrometry are shown. For the full list of identified proteins see Table S2.

To analyze phosphorylation of Swi5 and Sfr1 during meiosis, we used the TAP protocol to purify Sfr1-TAP together with associated proteins from meiotically induced *S. pombe* cells and analyzed the post-translational modifications by mass spectrometry. We found that Sfr1 was phosphorylated on serines S26, S33, S109 and S155. To identify additional phosphorylation sites, we mutated the five serine residues found to be phosphorylated in our Sfr1-TAP purifications (S26, S33, S109, S155 and S165) to alanine, which can no longer be phosphorylated (*sfr1-5A*) and purified Sfr1-5A-TAP from haploid meiotic cells. We found that Sfr1-5A was phosphorylated on S52 (or S48), T73, S135, S147 (or T146), T152, S175 and S253 (Figure S1, Table S1). In parallel, we purified Sfr1-TAP from diploid meiotic cells and found that Swi5 was phosphorylated on serine 84 and Sfr1 was phosphorylated on 13 residues (S26, S33, S52 (or S48), T73, S109, T114 (or S116), S135, S147 (or T146), T152 (or T151), S155, S165, S175 and S253) (Figure 1 and Table S2). Four Sfr1 single phosphorylation sites (S52 (or S48), T114 (or S116), S147 (or T146) and T152 (or T151)) could not be assigned to an individual amino acid. Most of the identified phosphorylation sites map to the N-terminal part of Sfr1, which is intrinsically disordered. Only Swi5 S72, S84 and Sfr1 S253 are visible on the available Swi5-Sfr1C structure (Figure S2).

Mass spectrometry analysis of proteins co-purifying with Sfr1-TAP revealed that, similarly as in cycling cells, Sfr1-TAP associated with high levels of Swi5 (Tables S1 and S2). Our identification of S84 phosphorylation on Swi5 that co-purified with Sfr1-TAP suggests that phosphorylated Swi5 is part of the Swi5-Sfr1 complex. We also found that Sfr1-5A-TAP but not Sfr1-TAP co-purified with small amounts of Dmc1, a meiosis specific member of the RecA-family of recombinases, suggesting that the interaction between the Swi5-Sfr1 complex and Dmc1 is transient, substoichiometric or easily disrupted (Tables S1 and S2). We conclude that during meiosis, Swi5 associates with Sfr1-TAP and both Swi5 and Sfr1 proteins are phosphorylated.

### 3.2. Phosphomimetic swi5 and sfr1 Mutants Are Not Fully Functional

To analyze the potential functional significance of Swi5 and Sfr1 phosphorylation, we generated *swi5* mutants encoding alanine at two identified phosphorylation sites S72 and S84 (*swi5-2A*) or the phosphomimetic aspartate at those positions (*swi5-2D*). Similarly, we mutated 13 identified Sfr1 phosphorylation sites (S26, S33, S52, T73, S109, T114, S135, S147, T152, S155, S165, S175 and S253) and generated *sfr1-13A* and *sfr1-13D* mutants.

**Figure 2:**
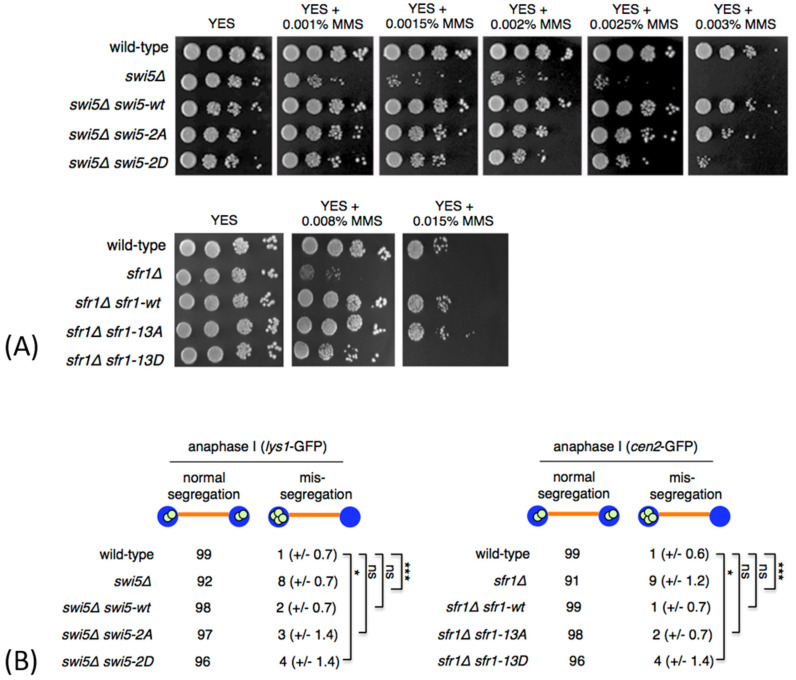
Phenotype of non-phosphorylatable and phosphomimetic *swi5* and *sfr1* mutants. (**A**) The indicated strains were grown on YES medium, diluted in 10-fold steps, spotted onto YES plates containing the indicated amounts of methyl methanesulfonate (MMS) and incubated for 3 days at 30 °C. (**B**) The cells were sporulated, fixed and immunostained for tubulin and GFP. DNA was visualized by DAPI. Segregation of chromosome I was scored in a wild type and *swi5* mutant strains carrying chromosome I marked by *lys1*-GFP. Segregation of chromosome II was scored in a wild type and *sfr1* mutant strains carrying chromosome II marked by *cen2*-GFP. *lys1*-GFP and *cen2*-GFP dots were scored under the fluorescence microscope in 100 anaphase I cells. Means ± standard deviations are shown. Unpaired *t*-test was performed for statistical analysis (*** *p* < 0.001; * *p* < 0.05; ns *p* > 0.05).

To test whether the Swi5 and Sfr1 residues that we found to be phosphorylated are important for the repair of damaged DNA, we treated cells with MMS to induce DNA lesions in vegetative cells [30]. While no increased MMS sensitivity was observed in *swi5-2A* and *sfr1-13A* mutants, phosphomimetic *swi5-2D* and *sfr1-13D* mutants were more sensitive to MMS, as compared to wild type (Figure 2A). While expression of a wild type Swi5 (*swi5*Δ *swi5-wt*) and Swi5-2A mutant (*swi5*Δ *swi5-2A*) rescued the MMS sensitivity phenotype of the *swi5*Δ mutant, mutant Swi5 protein carrying S72D and S84D substitutions only partially rescued the phenotype of the *swi5*Δ mutant (*swi5*Δ *swi5-2D*) (Figure 2A). Similarly, expression of a wild type Sfr1 (*sfr1*Δ *sfr1-wt*) and Sfr1-13A mutant (*sfr1*Δ *sfr1-13A*) rescued the MMS sensitivity phenotype of the *sfr1*Δ mutant, while Sfr1-13D mutant only partially rescued the phenotype of the *sfr1*Δ mutant (*sfr1*Δ *sfr1-13D*) (Figure 2A).

Swi5 and Sfr1 are also important for proper segregation of chromosomes during meiosis [5,31]. In order to analyze chromosome segregation, we introduced *swi5* mutations into a homothallic *h^90^* strain where chromosome I was marked with GFP (*lys1*-GFP) [32]. In this strain, GFP-tagged LacI molecules bind to *lacO* repeats inserted within the *lys1* locus located near the centromere. This strain generates cells of both mating types and undergoes mating and meiosis on a sporulation medium. Similarly, we introduced *sfr1* mutations into *h^90^* strain where chromosome II was marked with GFP (*cen2*-GFP) [33]. During anaphase I, homologous centromeres in wild-type cells segregated to opposite poles. However, we frequently observed homolog non-disjunction in *swi5*Δ and *sfr1*Δ anaphase I cells (Figure 2B). While expression of a wild-type Swi5 (*swi5*Δ *swi5-wt*) and Swi5-2A mutant (*swi5*Δ *swi5-2A*) rescued the missegregation phenotype of the *swi5*Δ mutant, Swi5-2D mutant protein only partially rescued the phenotype of the *swi5*Δ mutant (*swi5*Δ *swi5-2D*) (Figure 2B). Similarly, expression of a wild type Sfr1 (*sfr1*Δ *sfr1-wt*) and Sfr1-13A mutant (*sfr1*Δ *sfr1-13A*) rescued the missegregation phenotype of the *sfr1*Δ mutant, while Sfr1-13D mutant only partially rescued the phenotype of the *sfr1*Δ mutant (*sfr1*Δ *sfr1-13D*) (Figure 2B).

**Figure 3:**
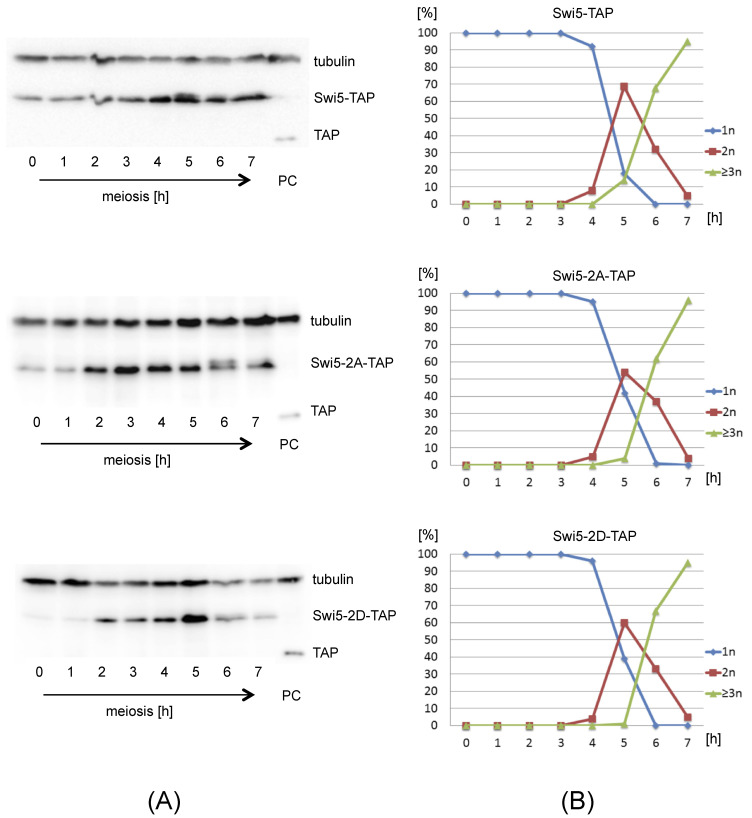
Expression of Swi5-TAP, Swi5-2A-TAP and Swi5-2D-TAP proteins during meiosis. (**A**) *pat1-114* cells expressing Swi5-TAP, Swi5-2A-TAP or Swi5-2D-TAP were arrested by nitrogen starvation and released into meiosis at 34 °C by inactivation of Pat1. Cells were harvested at the indicated time points (hours). Proteins extracted from meiotic cells were analyzed by gel electrophoresis and Western blotting using anti-tubulin antibodies. The TAP epitope was detected using PAP antibodies (rabbit antiperoxidase antibody linked to peroxidase). As a positive control (PC), protein extracts were prepared from a pool of cells expressing TAP tag alone, harvested at 4–6 hours after meiosis induction [36]. Full original images of Western blots are shown in Figure S4. (**B**) Progression of *pat1*-induced meiosis. Cells as described in (**A**) were harvested at the indicated time points (hours). Fixed cells were stained with DAPI and nuclei were counted in 100 cells per time point. Shown are the fractions of cells that contained one nucleus (1n), two nuclei (2n) or more than two nuclei (>3n) at the indicated time points.

The observed phenotype of phosphomimetic mutants *swi5-2D* and *sfr1-13D* is not due to lack of expression, as all four mutant proteins (Swi5-2A-TAP, Swi5-2D-TAP, Sfr1-13A-TAP and Sfr1-13D-TAP) were expressed, as detected by Western blot analysis of TAP-tagged proteins (Figure 3, Figures S3 and S4). The Western blot analysis showed that protein levels of both Swi5-TAP and Sfr1-TAP increase during meiosis, which is consistent with previous observation that *swi5* and *sfr1* mRNAs are upregulated during meiosis [34].

Taken together, we showed that Swi5 co-purifies with Sfr1-TAP isolated from meiotic cells. Our mass spectrometry analysis identified new phosphorylation sites on Swi5 and Sfr1 proteins. However, we found that the identified phosphorylation sites are dispensable for proper segregation of chromosomes during meiosis I and for repair of damaged DNA, as assessed by an MMS sensitivity test. While our results show that the absence of phosphorylation on the analyzed residues is not essential for the function of Swi5 and Sfr1, the phenotype of phosphomimetic mutations is difficult to interpret. Although phosphomimetic mutations are often used to mimic the constitutively phosphorylated state, it is known that the chemical environment created by phosphorylation is different from that of negatively charged amino acids [35]. Therefore, the potential functional relevance of Swi5 and Sfr1 phosphorylation remains to be determined.

## Supplementary Materials

Supplementary materials can be found at https://www.mdpi.com/article/10.3390/genes12071014/s1. Figure S1. Sfr1 phosphorylation sites identified by mass spectrometry. Figure S2. Visualization of Swi5 and Sfr1 phosphorylation sites. Figure S3. Expression of Sfr1-TAP, Sfr1-13A-TAP and Sfr1-13D-TAP proteins during meiosis. Figure S4. Full original images of Western blots shown in Figure 3. Table S1. Mass spectrometry analysis of Sfr1-5A-TAP. Table S2. Mass spectrometry analysis of Sfr1-TAP. Table S3. *S. pombe* strains and genotypes.

## Abbreviations

SDS-PAGE	sodium dodecyl sulfate–polyacrylamide gel electrophoresis
YES	yeast extract with supplements medium
EMM	Edinburgh minimal medium
MMS	methyl-methanesulfonate
CBP	calmodulin binding peptide
TAP	tandem affinity purification
