# Supplementary material for: Mapping and Analysis of Swi5 and Sfr1 Phosphorylation Sites
Source: Genes (Basel). 2021 Jun 30;12(7):1014. doi: 10.3390/genes12071014 (PMC8305525; doi:10.3390/genes12071014)
Supplement: Supplementary file 1 [file genes-12-01014-s001.zip › Figures_S1,S2,S3,S4.pdf]

Figure S1

A

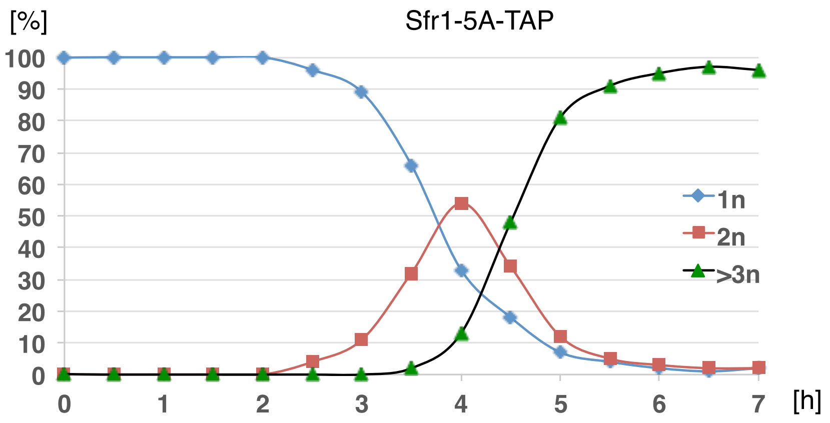

B

Sfr1-5A-TAP

	unique peptides	coverage	phosphorylated residues
Sfr1-5A (299 amino acids)	24	82%	S52 (or S48), T73, S135, S147 (or T146), T152, S175, S253
Swi5 (85 amino acids)	7	60%	

Figure S1. Sfr1 phosphorylation sites identified by mass spectrometry.
(A) Haploid *pat1-114* cells expressing Sfr1-5A-TAP were arrested by nitrogen starvation and released into meiosis at 34°C by inactivation of Pat1. Small aliquots of the cell culture were harvested at the indicated time points (hours). Fixed cells were stained with DAPI and nuclei were counted in 100 cells per time point. Shown are the fractions of cells that contained one nucleus (1n), two nuclei (2n) or more than two nuclei (>3n) at the indicated time points.
(B) The cells were harvested around 3 hours after meiosis induction and Sfr1-5A-TAP was isolated by tandem affinity purification. Purified proteins were analyzed by mass spectrometry. Phosphorylation sites identified on Sfr1 by mass spectrometry are shown. For the full list of identified proteins see Table S1.

Figure S2

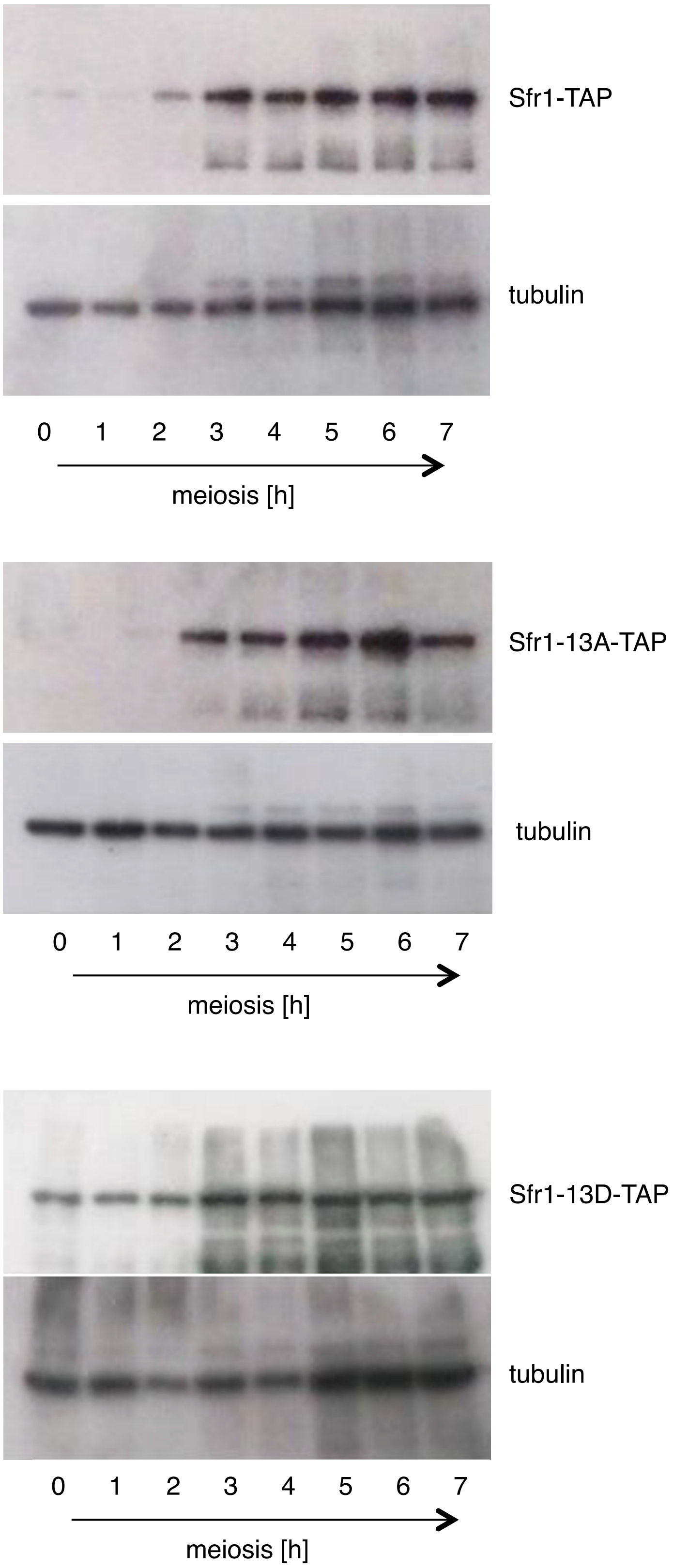

Figure S2. Expression of Sfr1-TAP, Sfr1-13A-TAP and Sfr1-13D-TAP proteins during meiosis.
pat1-114 cells expressing Sfr1-TAP, Sfr1-13A-TAP and Sfr1-13D-TAP were arrested by nitrogen starvation and released into meiosis at 34°C by inactivation of Pat1. Cells were harvested at the indicated time points (hours). Proteins extracted from meiotic cells were analyzed by gel electrophoresis and Western blotting using anti-tubulin antibodies. The TAP epitope was detected using PAP antibodies (rabbit antiperoxidase antibody linked to peroxidase).

Figure S3

A

Swi5

```
Sc (Sae3)   ------------------------------------------------------------   0
Sp          ------------------------------------------------------------   0
Mm          ------------------------------------------------------------   0
Hs          MQRRGQRDLWRHNKSCARNRCPRPPRERGGAGFPWVRAQLSVRQFTLRVRVPGPVHLRGR   60

Sc (Sae3)   ------------------------------------------------------------   0
Sp          ------------------------------------------------------------   0
Mm          ------------------------------------------------------------   0
Hs          SPTPALDPLAPLNPLIRGPRTPGLRRWIQSLALLLPNCSSSRIPTVPRPHSGLWVQSDFP   120

Sc (Sae3)   -------------------------------MNYLETQLNKKQKQIQEYE-SMNGNLIKM   28
Sp          ---------------------------------MEKSQLESRVHLLEQQKEQLESSLQDA   27
Mm          --------------------------MIDENNDVSEEALSSDIKKLKEKHDMLDKEISQL   34
Hs          LGFLSRTEPRLTRSCRGAFRSPRPLPKSGQADGTSEESLHLDIQKLKEKRDMLDKEISQF   180
                                               :  *    : ::: .  :: .: .

Sc (Sae3)   FEQLSKEKKNDETPKKISSTYIKELKEYNELRDAGLRLAQIIADEKQCKIKDVFEEIGYS   88
Sp          LAKLKNRD-----AKQTVQKHIDLLHTYNEIRDIALGMIGKVAEHEKCTSVELFDRFGVN   82
Mm          ---IAEGY-----RVIELEKHISLLHEYNDIKDVSQMLLGKLAVTRGVTTKELYPDFDLN   86
Hs          ---VSEGY-----SVDELEDHITQLHEYNDIKDVGQMLMGKLAVIRGVTTKELYPEFGLD   232
               : :            . :*  *: **:::* .  :   :*  .  .  :::  :. .

Sc (Sae3)   MKD      91
Sp          GSE      85
Mm          LND      89
Hs          MND      235
             .:
```

Sfr1

```
Sc (Mei5)   ------------------------------------------------------------   0
Sp          MSQTINSELNENATSQCKEDLKVSLSESDLRDSQGQLGIENPPKCNNS---GNHSDNLGF   57
Mm          ----MAEEGNQEFTSKMENSS-----DSASTSPDAPQPSENPPSPPTSPAAPQTSENPPS   51
Hs          ---MAEGEKNQDFTFKME------------------------------------------   15

Sc (Mei5)   ------------------------------------------------------------   0
Sp          IEQSETVHPENEKA-------LTPDLRDT-------------KIHTSLPITT--------   89
Mm          PPTSPAVPQTRENPPSPPTSPAAPQPRENPPSPPTSPAAPQPRENPPSPPTSPAAPQPRE   111
Hs          -------------------------------------------SPSDSAVVLPSTPQASA   32

Sc (Mei5)   ------------------------------------------------------------   0
Sp          -PFSKKRAREAKNILLKPFKSPLRQTASPQVADTNLKPSLAVTNLNSDETNTSSEPVTSP   148
Mm          NPPSPHSNSSGKQPLSGTPKERLKKARSSSHSFCSVVKRMKVENDENN--ETLSEPGESS   169
Hs          NPSSPYTNSSRKQPMSATLRERLRKTRFSFNSSYNVVKRLKVESEEND--QTFSEKPASS   90

Sc (Mei5)   -----------MHNQEEWLDKDKTL--VNEEENTCINHSYTKKDTNNYRVGKSGIKD-LK   46
Sp          LRT----TPNSIKRQKRL------F---KSPISNCLNPKSDP-----------EITQLLS   184
Mm          KEENCSKAQESLKNKDSEPGEKS-----SEEKNTCESKSSDTGSSNALPKES-E-NAIIR   222
Hs          TEENCLEFQESFKHIDSEFEENTNLKNTLKNLNVCESQSLDSGSCSALQNEF-VSEKLPK   149
                       ::. .             .  . * . .

Sc (Mei5)   KPTNQKEIAIKNRELTKQLTLLRQENNHLQQACKILSENKIIENRKSIEKWRTICEMELS   106
Sp          R-----R-----LKLEKEVRNLQEQLITAETARKVEAKNEDKDLQTLIQKWKNAAQQAAE   234
Mm          EKLKQEK-----IRLIRQVEEKEDLLRRLKLVKMYRIKNDVTELENLIKKWRKCGQRLLC   277
Hs          QRLNAEK-----AKLVKQVQEKEDLLRRLKLVKMYRSKNDLSQLQLLIKKWRSCSQLLLY   204
            .     .      .* :::   .:     : .     :*.  : .  *:**:.  :

Sc (Mei5)   FILNSTLIKINRMGGYKDFLEKEMEAKKRR---------L-EYQIDNGMEDQICEIKESD   156
Sp          VLFKPMAERIRLAGGVTQSFRIEEGENKGQIQEVRTEFTMSMFLNQFGVPVHLMSFDE--   292
Mm          ELQSIMSED------------------------EDEKLTLTELIDFYGIDDNLLHYNR--   311
Hs          ELQSAVSE-------------------------ENKKLSLTQLIDHYGLDDKLLHYNR--   237
             : .                                   :       *:  ::   ..

Sc (Mei5)   DFRQLSEVEKQEWESQMNEQLKELEKKKIAELEKLNKVLHDSEGKDFGMAELCTRLKLDY   216
Sp          --------ENGDWKS---------------------------------------------   299
Mm          --------SEEEFTGV--------------------------------------------   319
Hs          --------SEEEFIDV--------------------------------------------   245
                    .: :: .

Sc (Mei5)   SLIFPQ   222
Sp          ------   299
Mm          ------   319
Hs          ------   245
```

B

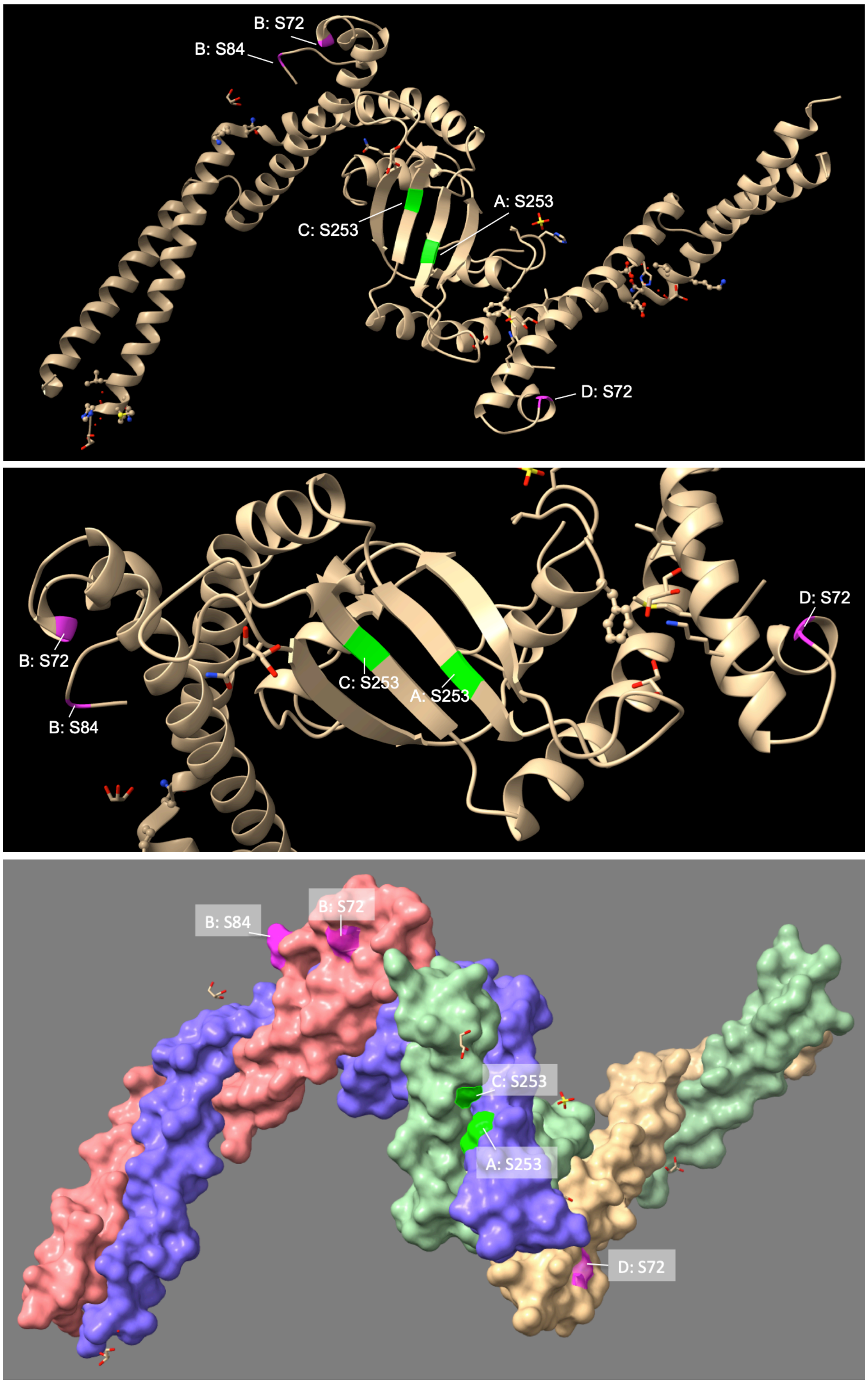

B: S72
B: S84
C: S253
A: S253
D: S72
B: S72
B: S84
C: S253
A: S253
D: S72
B: S84
B: S72
C: S253
A: S253
D: S72

Figure S3. Visualization of Swi5 and Sfr1 phosphorylation sites.
(A) Swi5 and Sfr1 protein sequences from *Saccharomyces cerevisiae* (Sc), *Schizosaccharomyces pombe* (Sp), *Mus musculus* (Mm) and *Homo sapiens* (Hs) were aligned using Clustal Omega (Sievers et al., 2017; https://pubmed.ncbi.nlm.nih.gov/21988835). An asterisk (*) indicates positions of fully conserved residues, a colon (:) indicates conservation between residues of strongly similar properties and a period (.) indicates conservation between residues of weakly similar properties. Swi5 and Sfr1 phosphorylation sites are highlighted in red.
(B) Crystal structure of fission yeast Swi5 and its complex with the Sfr1C (C-terminal domain, residues 181-299) was retrieved from the RCSB Protein Data Bank (PDB ID: 3VIQ) (Kuwabara et al., 2012; https://pubmed.ncbi.nlm.nih.gov/22405003) and visualized using ChimeraX software (Pettersen et al., 2021; https://pubmed.ncbi.nlm.nih.gov/32881101). Presented are the two Swi5-Sfr1C heterodimers in the asymmetric unit. Swi5 and Sfr1 phosphorylation sites located on individual protein chains (denoted A, B, C and D) are indicated in magenta and green, respectively. Upper and middle figures: ribbon diagram of Swi5-Sfr1C, lower figure: surface representation of Swi5-Sfr1C.

Figure S4

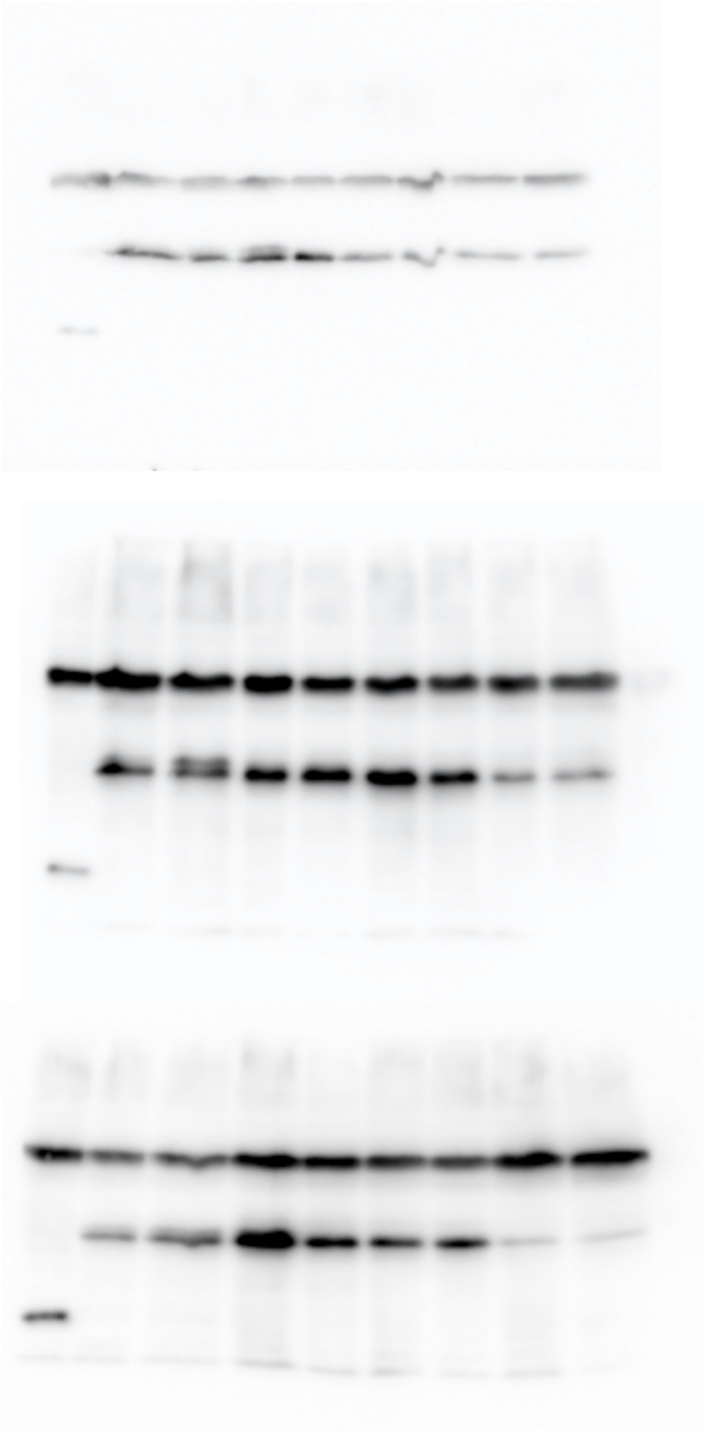

Figure S4. Full original images of Western blots shown in Figure 3.
