# Supplementary material for: Mapping and Analysis of Swi5 and Sfr1 Phosphorylation Sites
Source: Genes (Basel). 2021 Jun 30;12(7):1014. doi: 10.3390/genes12071014 (PMC8305525; doi:10.3390/genes12071014)
Supplement: Supplementary file 1 [file genes-12-01014-s001.zip › TableS3.pdf]

**Table S3. *S. pombe* strains and genotypes**

| Strain number | Genotype | Used in |
|---|---|---|
| JG16489 | *pat1-114 sfr1-5A-(S26A, S33A, S109A, S155A, S165A)-TAP::KanMX6* | Figure S1, Table S1 |
| JG15195 | *$h^{-}$/$h^{-}$ ade6-210/ade6-216 pat1-114/pat1-114 sfr1-TAP::KanMX/sfr1-TAP::KanMX6* | Figure 1, Table S2 |
| JG12618 | *$h^{90}$ ade6-216 leu1-32 lys1-131 ura4-D18 cen2(D107)::kan-$ura4^{+}$-lacO $his7^{+}$::lacI-GFP* | Figure 2 |
| JG15974 | *$h^{90}$ ade6-216 leu1-32 lys1-131 ura4-D18 cen2(D107)::kan-$ura4^{+}$-lacOp $his7^{+}$::lacI-GFP sfr1::NatMX4* | Figure 2 |
| JG17984 | *$h^{90}$ ade6-216 leu1-32 lys1-131 ura4-D18 cen2(D107)::kan-$ura4^{+}$-lacOp $his7^{+}$::lacI-GFP sfr1::NatMX4 $sfr1^{+}$::hphMX4* | Figure 2 |
| JG17985 | *$h^{90}$ ade6-216 leu1-32 lys1-131 ura4-D18 cen2(D107)::kan-$ura4^{+}$-lacOp $his7^{+}$::lacI-GFP sfr1::NatMX4 sfr1-13A::hphMX4* | Figure 2 |
| JG17986 | *$h^{90}$ ade6-216 leu1-32 lys1-131 ura4-D18 cen2(D107)::kan-$ura4^{+}$-lacOp $his7^{+}$::lacI-GFP sfr1::NatMX4 sfr1-13D::hphMX4* | Figure 2 |
| JG11248 | *$h^{90}$ leu1 ura4 LacO-$lys1^{+}$ GFP-LacI-$his7^{+}$* | Figure 2 |
| JG14998 | *$h^{90}$ leu1 ura4 LacO-$lys1^{+}$ GFP-LacI-$his7^{+}$ swi5::NatMX4* | Figure 2 |
| JG17812 | *$h^{90}$ leu1 ura4 LacO-$lys1^{+}$ GFP-LacI-$his7^{+}$ swi5::NatMX4 $swi5^{+}$::hphMX4* | Figure 2 |
| JG17813 | *$h^{90}$ leu1 ura4 LacO-$lys1^{+}$ GFP-LacI-$his7^{+}$ swi5::NatMX4 swi5-2A::hphMX4* | Figure 2 |
| JG17814 | *$h^{90}$ leu1 ura4 LacO-$lys1^{+}$ GFP-LacI-$his7^{+}$ swi5::NatMX4 swi5-2D::hphMX4* | Figure 2 |
| JG17933 | *pat1-114 leu1 ura4 LacO-$lys1^{+}$ GFP-LacI-$his7^{+}$ swi5::NatMX4 $swi5^{+}$::hphMX4 swi5-TAP::kanMX6* | Figure 3, Figure S4 |
| JG17934 | *pat1-114 leu1 ura4 LacO-$lys1^{+}$ GFP-LacI-$his7^{+}$ swi5::NatMX4 swi5-2A::hphMX4 swi5-2A-TAP::kanMX6* | Figure 3, Figure S4 |
| JG17935 | *pat1-114 leu1 ura4 LacO-$lys1^{+}$ GFP-LacI-$his7^{+}$ swi5::NatMX4 swi5-2D::hphMX4 swi5-2D-TAP::kanMX6* | Figure 3, Figure S4 |
| JG17886 | *$h^{+}$/$h^{+}$ ade6-M210/ade-M216 pat1-114/pat1-114 meu5::TAP-kanMX/pat1-114 meu5::TAP-kanMX6* | Figure 3 (PC), Figure S4 |
| JG18174 | *$h^{-}$ pat1-114 ade6-210 sfr1::NatMX4 $sfr1^{+}$::hphMX4 sfr1-TAP::kanMX6* | Figure S2 |
| JG18177 | *$h^{-}$ pat1-114 ade6-210 sfr1::NatMX4 sfr1-13A::hphMX4 sfr1-13A-TAP::kanMX6* | Figure S2 |
| JG18204 | *$h^{-}$ pat1-114 ade6-210 sfr1::NatMX4 sfr1-13D::hphMX4 sfr1-13D-TAP::kanMX6* | Figure S2 |

(other auxotrophic markers not scored)
